# Correlates of conduct disorder among inmates of a Nigerian Borstal Institution

**DOI:** 10.1186/s13034-016-0100-0

**Published:** 2016-06-21

**Authors:** Anthony Ademola Olashore, Adegboyega Ogunwale, Timothy Olaolu Adebowale

**Affiliations:** Department of Psychiatry, Faculty of medicine, University of Botswana, Private Bag 00713, Gaborone, Botswana; Neuro-psychiatry Hospital, PMB, 2002, Aro, Abeokuta, Ogun State Nigeria

**Keywords:** Correlates, Conduct disorder, In-mates, Nigerian, Borstal Institution

## Abstract

**Background:**

Juvenile delinquency has become a significant global problem. Conduct disorder (CD), among other psychiatric disorders, has assumed prominence in its association with juvenile offending as well as criminality in adulthood. Despite this knowledge, little attention is given to this problem especially as it affects adjudicated adolescent offenders in developing countries.

**Aim:**

To examine the prevalence and correlates of CD among incarcerated adolescents in a Nigerian Borstal Institution and to investigate its independent predictors.

**Methods:**

A cross-sectional descriptive study was conducted among 147 inmates of a Borstal Institution in Abeokuta, South Western Nigeria. A self-administered questionnaire and interviewer administered MINI-KID were used. The associations between conduct disorder and socio demographic as well as forensic variables were investigated using Chi square statistics while logistic regression was used to predict CD.

**Results:**

Out of 147 respondents, 83 (56.5 %) met the criteria for CD with a mean age 17.1 ± 1.1. Of the socio-demographic and forensic variables investigated, number of siblings (OR 4. 630; p = 0.010; 95 % CI 1.433–14.964) and previous history of incarceration (OR 4. 99; p = 0.043; 95 % CI 1.048–23.846) emerged as independent predictors of CD.

**Conclusions:**

This study recorded a high prevalence of conduct disorder among a sample of incarcerated juvenile offenders. The association of conduct disorder with large family size and recidivism highlights the need for comprehensive early interventions focused on improving parental supervision in large families as well as other re-training programs aimed at reducing juvenile re-offending.

## Background

Parents had long worried over what to do with children considered to be ‘beyond parental control’ several decades before conduct disorder (CD) became a medical diagnosis in late sixties [1]. It has now become one of the most frequently diagnosed psychiatric condition in present-day child psychiatry [2].

Conduct disorder is a complicated group of behavioral problems in children and adolescents, which is characterized by “repetitive and persistent pattern, in which the basic rights of others or major age-appropriate societal norms or rules are violated” [2, 3]. According to DSM IV, CD has been described as a disorder often characterized by a pervasive and persistent pattern of aggressive, deceptive, and destructive behavior that usually begins in childhood or adolescence [2, 3]. Behaviors exhibited by children with CD include aggression towards people or animals; destruction of properties; deceitfulness; drug and alcohol use, theft and other delinquent/disorderly behaviors [3].

The two major classification systems, i.e., ICD and DSM, require the presence of at least 3 of 15 symptoms over 6 months duration for diagnosis, and it is commoner in males than female [2, 3]. DSM has an additional category of disruptive behavioral disorder, ‘oppositional defiant disorder’ (ODD), for individuals with persistent hostility, defiant, provocative and disruptive behavior which is out of the normal range, but without aggressiveness and antisocial behavior [2]. Nevertheless, this current study focuses only on CD and its correlates.

Several cross-national studies have suggested that more than 60 % of youths in the detention of juvenile justice and 50–90 % of children and adolescents in various residential/foster care [4–7], have a diagnosable psychiatric disorder compared to 15–25 % of the general population [2–4, 8]. Psychiatric disorders ranging from mood disorders, psychosis, anxiety disorders, ADHD, disruptive behavioral disorder and substance abuse disorder had been widely described in these settings. Of these psychiatric disorders, CD commands some level of prominence in not only showing an association with offending but also predicting adult criminality later in life and persistence of other co-morbid mental disorders [9, 10]. Thus, as there is much evidence that CD is a precursor of criminality (and antisocial personality disorder), there is a strong need for interventions in childhood and adolescence [9–11].

Authors have suggested that the prevalence of CD is particularly high in forensic settings than in the general population [2–4]. A prevalence rate of 41 % was reported among 1829 young offenders in the United States, compared to (5–16 %) in the general population [2, 4]. A systematic review and meta-regression analysis of 25 surveys across Europe and America also revealed a prevalence rate of 52.8 % [7]. In the United Arab Emirates, a prevalence of 24.7 % was reported among 72 incarcerated offenders compared to the 7 % found among school children [12, 13], whereas, in Brazil, 77 % prevalence rate was reported among 116 juvenile delinquents [14]. Although, data from Africa are still very scanty, a study by Adegunloye et al. [5] reported a 60 % prevalence rate among 58 incarcerated juveniles in the North-central part of Nigeria, compared to 15.8 % prevalence rate reported among a sample of Nigerian school children [8].

On a psychoanalytical plane, this behavioral disorder has been ascribed to “unresolved conflicts between the psychic elements of id, ego and super-ego,” or, an expression of a generally “disturbed” lifestyle [15]. Currently, CD has been suggested to arise from a complex combination of risk factors which include biological and environmental influences such as parental criminality, defective rearing, physical abuse and psychological distress. Other factors consistently associated with CD in many studies, including those carried out among non-offenders are low socio-economic status, family dysfunction [5, 12, 14, 17], large family [16, 17], as well as decline in frequency of religious practice [14, 18]. Drug related problems have also been implicated in many studies [4, 8, 14, 19, 20]. Whilst increase in frequency of religious practice was found to be associated with a reduced risk of offending [14, 18], low socio-economic status, family dysfunction, large family, amongst others were associated with increased risk [2, 3, 12, 14, 16, 17]. With the exception of large family size which may be a function of polygamous family setting often practiced in Africa and the Arabian countries [5, 12, 13, 17], most studies from Europe and America agree with the few African studies on the association of these risk factors and CD [4, 6, 7, 20].

The consequences of this disorder often have far-reaching consequences on the lives of these individuals, their families and the community at large [2, 3]. Apart from the social costs of CD at the individual, family and community levels, CD has a substantial economic impact, manifesting in terms of utilization of publicly resourced health and social services, as well as the judicial and penal systems [2, 3].

In spite of the significant association of CD with offending behavior and its socio-economic impact [3, 9, 10], there remains a dearth of knowledge on its prevalence and common risk factors in Nigeria [5]. This has a significant consequence on Mental Health services available for this group of people as well as service utilization by the parent of affected individuals [5]. On one hand, CD, which presents with offending as alluded to earlier, is often viewed by the government as more of a violation of societal norms and criminal acts than a mental illness that require medical attention. Consequently, punishment rather than quality mental health service is offered as remedy. In support of this claim, a report of a study by the committee on the right of the child revealed that, over two-third of the juvenile offenders who entered the juvenile justice system via the police suffered both physical and verbal abuse. About 45.9 % of those already within the system complained of being subjected to mental and physical torture, while another 30 and 31 % respectively reported being denied food and long detention period without trial [21]. This ultimately exposed them to more dangerous vices, rather than addressing their needs, thus strengthening the cycle of recidivism [15, 20].

On the part of the caregivers/parents, the dearth of research has continued to bread ignorance and poor service utilization [5, 22]. Faulty perception that children do not suffer from mental illness; fear of expression because of punishment and the continuing belief in spiritual possession with its attendant preference for spiritual/alternative method of treatment, are all products of ignorance. As a result, parents of affected children do not seek help [22]. They ultimately label these children, “Beyond parental control” and so either abandon them or send them to some “government correctional facilities” where they meet with more criminally minded children and learn more dangerous vices [15, 21].

Understanding CD as one of the major causes of delinquency has gross implication for the socio-legal treatment/rehabilitation of juvenile offenders. In addition, it will assist in health care planning and formulation of interventions towards prevention of delinquency as well as breaking the cycle of recidivism [19, 20]. As a step toward this, the current study sought to examine the prevalence and correlates of CD using a slightly larger sample size than was used previously in forensic settings in Nigeria [5, 23] and to investigate which of these correlates predict CD.

## Methods

### Setting of the study

A cross-sectional descriptive study was conducted among the in-mates of the Borstal Institution in Abeokuta, South Western Nigeria. The Borstal Institution which was established in 1984 is one of the three Borstal Institutions in Nigeria. The other two are in Ilorin and Kaduna both in the northern part of Nigeria. This facility is under the management of the Nigerian Prison Service, and is run by a senior prison officer who serves as Principal. Though a restrictive environment, it operates as a correctional facility with emphasis on educational and vocational training for offenders incarcerated therein. However, the available facilities are inadequate to effectively meet the needs of these inmate.

### Recruitment strategy

One hundred and ninety (190) in-mates were met in the institution at the time of study. Of these, 20 were already above 18 years as at their last birthday and so were excluded from the study because of the suitability of the Mini-Kid instrument [24]. The remaining 170 were gathered and the study and its purpose was explained to them. Six (6) of the remaining 170 in-mates refused to give their consent, another 16 were further excluded due to communication problems and only 148 in-mate who agreed to participate were finally interviewed.

### Study design and instruments

This is a cross-sectional descriptive study using two sets of instruments: The socio-demographic questionnaire which was designed by the researcher based on the previous studies [4, 5, 23] and Mini International Neuropsychiatric Interview for Children and Adolescents (MINI-KID).

MINI-KID is a structured clinical diagnostic interview designed to assess the existence of current ICD-10 and DSM-IV mental disorders in children and adolescents in a comprehensive and concise way [24]. It has been used in Nigeria by Adegunloye [5]. It is organized in diagnostic sections or modules for various psychiatric disorders such as psychotic disorders, affective disorders, CD etc. CD is coded with “P” and it has three parts; the P1 which is the screening question that asks whether there has been any complaint about the respondent, either from his teachers, friends, or parents. It is only when the response to P1 is “YES” that the interviewer will proceed to the next part. The second part, i.e., P2 is the main part of the module and has fifteen (15) questions, itemized “a–o”. This part of conduct disorder module asks question about the behaviors needed to make a diagnosis of conduct disorder. The last part which is coded P3 has only one (1) question. This part asks whether the behaviors elicited in P2 has caused a big problem either at school, home, within the family or friends. The interviewer is expected to circle (code) either “YES” or “NO” according to the response of the person being interviewed. To meet the criteria for conduct disorder, three (3) or more questions must be coded “YES” in P2, and P3 must also be coded “YES”.

Both instruments were translated into Yoruba (the predominant language in the south-western Nigeria). The socio-demographic section was self-administered, while the MINI-KID was administered by the researcher. The socio-demographic questionnaire was administered in 1 day. Each of the participants was given a number corresponding to the number on his questionnaire. This was done in order to be able to combine the two parts of the questionnaire during collation. The interview was not conducted every day, because of other activities on their schedule.

### Ethical approval

Ethical approval was obtained from the Health Research Ethics Committee of the Neuropsychiatric Hospital, Aro, Abeokuta, Nigeria. Permission to conduct the research was also sought from the authorities of the Nigeria Prison Service and a written consent was obtained from every respondent in accordance with the state law after explaining the whole procedure as well as the purpose of the study.

### Data analysis

The statistical analysis was done using Statistical Package for Social Sciences (SPSS) for window-version 16.0. Frequency tables were used for descriptive statistics and cross tabulations for relationship between variables. The associations between Conduct disorder and nominal variables such as religious participation, parental marital status, etc., were investigated using Chi square statistics. The variables that were significantly associated with CD on bivariate analysis were further entered into stepwise binary logistic regression analysis with backward elimination with the presence or absence of CD as the dependent variable. A p value of less than 0.05 was accepted as the level of statistical significance.

## Results

One hundred and forty-eight inmates participated in the study. Of the 148 questionnaires administered, one was excluded from analysis due to inadvertent loss of a page during collation. A total number of 147 questionnaire booklets were finally analyzed.

### Socio-demographic characteristics (Table 1)

All the participants were within the age range of 14–18 years. The mean age of the participants in years was 17.1 (SD 1.1). Majority of the respondents were from the main ethnic group in the part of the country in which the study was conducted (Yoruba ethnic group), representing 77.6 % of the total number of the participants. Christianity was the predominant religion (58.2 %) followed by Islam (40.1 %). More participants (58.5 %) practiced their religion less frequently, of the 147 in-mates. The highest level of education attained by the participant was the Senior Secondary School Certificate and only a quarter (25.2 %) of them were educated up to this level. Only 96 (65.3 %) of the participant reported that both of their parents are alive, of these, 63 (65 %) were divorced/separated, 7 (7.3 %) were living apart but still married/never married, while 26 (27.1 %) were married and living together. One hundred and twenty-one (82.3 %) of the in-mates grew up with a single parent or relatives and 24.5 % had been previously remanded.

**Table 1:** Socio-demographic characteristics

Characteristic	Mean (SD)
Age (in years)	17.1 (1.1)
Age range	14–18 years

	n (%)
Ethnicity	147 (100)
Yoruba	114 (77.6)
Hausa	5 (3.4)
Ibo	27 (18.4)
Other	1 (0.4)
Type of religion	147 (100)
African traditional religion	1 (0.7)
Christianity	87 (59.2)
Islam	59 (40.1)
Frequency of religious participation	147 (100)
Rarely or never	86 (58.5)
Often or regularly	61 (41.5)
Educational level	147 (100)
No formal education	6 (4.1)
Primary education	53 (36.1)
Junior secondary	51 (34.7)
Senior secondary	37 (25.2)
No of siblings	147 (100)
Small (four or less)	56 (38.1)
Large (five or more)	91 (61.9)
Who brought you up?	147 (100)
Single parent or relative	121 (82.3)
Both parents	26 (17.7)
Age (years) at parental separation/divorce/death	121 (100)
Less than five	92 (76.0)
Five and above	29 (24.0)
Family settings	147 (100)
Monogamous	57 (38.8)
Polygamous	90 (61.2)
Marital status (if both parent are alive)	96 (100)
Married and living together	26 (27.1)
Married but not living together	7 (7.3)
Separated or divorced	63 (65.5)
Reason/s for being incarcerated	147 (100)
Offender	97 (66.0)
Non-offender	50 (34.0)
History of previous remand	147 (100)
No	111 (75.5)
Yes	36 (24.5)
Duration of stay in weeks (months)	147 (100)
<12	67 (45.6)
12–24	76 (51.7)
>24	4 (2.7)
Prevalence of conduct disorder (CD)	
Conduct disorder (CD)	147 (100)
CD present	83 (56.5)
CD absent	64 (43.5)

### Prevalence of conduct disorder (Table 1)

Based on the diagnostic criteria of MINI- KID which requires three (3) or more questions to be coded “YES” in P2, and P3, eighty-three of the one hundred and forty-seven respondents met the criteria for CD (56.5 %) while the remaining sixty-four (43.5 %) did not.

### Socio-demographic characteristics and CD (Table 2)

Majority of those who met the criteria for conduct disorder were 17 years and above (*χ*^*2*^ = 4.164, p = 0.041). Other variables that have significant relationships with CD include; family setting (*χ*^*2*^ = 5.276, p = 0.022); number of siblings (*χ*^*2*^ = 6.812, p = 0.009); parents’ marital status (*χ*^*2*^ = 14.668, p < 0.01); who brought the participant up (*χ*^*2*^ _=_ 17.812, p < 0.001); age at parental loss (*χ*^*2*^ = 4.362, p = 0.037) and previous history of incarceration (*χ*^2^ = 8.812, p = 0.003).

**Table 2:** Socio-demographic profile and conduct disorder

Variables	Absent N (%)	Present N (%)	Degree of freedom	Chi square	p value
Age group					
14–16	16 (25.0)	10 (12.0)	1	4.164	0.041^a^
17 and 18	48 (75.0)	73 (88.0)			
Ethnicity					
Yoruba	46 (71.9)	68 (81.9)	3	4.705	0.195
Hausa	4 (6.2)	1 (1.2)			
Ibo	13 (21.9)	13 (15.7			
Others	0(0)	1 (1.20)			
Religion					
Christianity	36 (56.2)	51 (61.4)	2	1.581	0.454
Islam	27 (42.2)	32 (38.6)			
African traditional religion	1 (1.6)	0 (0)			
Frequency religious participation					
Rarely or never	37 (57.8)	49 (59.0)	1	0.022	0.881
Often	27 (42.2)	34 (41.0)			
Family setting					
Monogamous	32 (50.0)	26 (31.3)	1	5.276	0.022^a^
Polygamous	32 (50.0)	57 (68.7)			
No of siblings					
Four or less	32 (50.0)	24 (28.9)	1	6.812	0.009^a^
Five or more	32 (50.0)	59 (71.1)			
Marital status (if both parents are alive)					
Divorced/separated	24 (51.1)	39 (79.6)	2	14.668	0.001^a^
Never married/Married but not living together	2 (4.3)	5 (10.2)			
Married and living together	21 (44.6)	5 (10.2)			
Who brought the participant up					
Both parents	21 (32.8)	5 (6.0)	1	17.812	<0.01^a^
Single parent/relatives	43 (67.2)	78 (94.0)			
Age at parental separation/divorce/death					
Less than 5 years	28 (65.1)	64 (82.1)	1	4.362	0.037^a^
5 years or more	15 (34.9)	14 (17.9)			
Previous history of incarceration					
No	56 (87.5)	55 (66.3)	1	8.812	0.003^a^
Yes	8 (12.5)	28 (33.7)			
Reason for being incarcerated					
Criminal	19 (29.7)	31 (37.3)	1	0.945	0.331
Non-criminal	45 (70.3)	52 (62.7)			
Duration of stay					
<12 months	32 (50.0)	35 (42.2)	2	1.279	0.528
12–24 months	31 (48.4)	45 (54.20			
>24 months	1 (1.6)	3 (3.6)			

^a^Significan test of association

### Predictors of conduct disorder on multivariate binary logistic regression analysis (Table 3: The first and the last step of the model)

In the multivariate logistic regression to determine which of the significant variables from bivariate analysis would independently predict CD: age range, ‘who brought the participant up’, family setting, number of siblings, parents’ marital status, age at parental loss and previous history of incarceration were included into the model. The first variable to drop out of the model was ‘who brought the participant up’ while variables such as age, family setting, parents’ marital status, age at parental loss did not significantly add to the prediction of CD. Two independent predictor variables, namely: number of siblings (OR 4. 630; p = 0.010; 95 % CI 1.433–14.964) and previous history of incarceration (OR 4. 99; p = 0.043; 95 % CI 1.048–23.846) were produced by the regression model. These two independent predictors explain 23.6 % of the variance in prevalence of CD (Nagelkerke R^2^ = 0.236).

**Table 3:** Predictors of conduct disorder as confirmed by multiple logistic regression analysis

Variables	Wald	df	p value	OR	95 % CI	
					Lower	Upper
Step 1						
Age group^a^	1.013	1	0.314	2.063	0.504	8.447
Marital status if both parent are alive^b^	0.320	1	0.571	0.552	0.71	4.312
Family setting^c^	0.029	1	0.866	1.132	0.269	4.773
Age at parental separation/divorce/death^d^	3.270	1	0.071	3.283	0.905	11.910
No of siblings^e^	3.433	1	0.064	3.943	0.924	16.829
Previous history of incarceration^f^	4.258	1	0.039	5.383	1.088	26.632
Step 4						
Age at parental separation/divorce/death^d^	3.395	1	0.065	3.259	0.927	11.450
No of siblings^e^	*6.558*	*1*	*0.010*	*4.630*	*1.433*	*14.964*
Previous history of incarceration^f^	*4.076*	*1*	*0.043*	*4.999*	*1.048*	*23.846*

Significant test of association in italics

^a^14–16

^b^Married and living together

^c^Monogamous setting

^d^5 years or more

^e^Four or less

^f^No history of previous incarceration

## Discussion

All the one hundred and forty-seven (147) participants were males, which is a direct consequence of the single-sex (only males) Borstal care system being practiced currently in Nigeria [5, 23], unlike what obtains in other parts of the world where there are facilities for both sexes [4, 7, 14]. The mean age of 17.1 ± 1.1 years agrees with what had been previously reported in forensic settings in Nigeria [5, 23] and majority (86.7 %) are from Yoruba ethnic group which is probably a reflection of the location of the facility.

Our study showed that more than half (56.5 %) of the incarcerated participants had CD; a rate comparable to those observed across Europe and America, in systematic review of 25 surveys (56.5 vs 52.8 %) [7], but slightly lower than what was reported by Adeguloye (64.2 %) [5] in Ilorin, North Central part of Nigeria. The current rate may either be a consequence of regional differences within the country or a result of the slightly larger sample size in our study, which obviously reduces the proportion of the actual number of in-mates meeting the diagnosis of CD. Nonetheless, it follows the trend that had been shown by earlier studies [4, 5, 7, 12, 14], which indicate that CD is several times higher in residential care settings than in the general population. In the current study for example, the prevalence rate (56.5 %) is three times as high as was reported in school children (15.6 %) in Nigeria [8].

The specific correlates of CD in this study revealed some interesting but disturbing trends. Although, with multiple regression analysis, older age did not contribute significantly to the prediction of CD (OR 821; p = 0.756; 95 % CI 0.236–2.851), we found a significant relationship between older and CD (*χ*^2^ = 4.164, p = 0.041). On this, our study diverges from the findings of Adegunloye [5], in a different part of the country and an American study, both of which indicated an association in the opposite direction [5, 24]. It is possible that variables such as duration of stay (54.4 % had spent >12 months) or repeated incarceration might have contributed to an older age of the affected adolescent in this study.

Religiosity (described as frequent participation in religious activities) with frequent teaching of retributive justice may lower the chance of being involved in antisocial practices, whereas, a lower level of religiosity might predispose to vices and criminal behavior, with consequent delinquency and ultimate incarceration [18, 25]. In our study, neither the type of religion (*χ*^2^ = 1.661, p = 0.197) nor religiosity (X = 0.002, p = 0.881) was significantly associated with a lower prevalence of CD. This was unlike the studies conducted in school children [18, 25] but in accord with those conducted among incarcerated juveniles [5, 12] where religious participation was not shown to be protective. This might suggest that religiosity was only associated with CD among non-incarcerated young population rather than in forensic settings wherein participation in religious activities might be an imposition rather than choice.

While our study does not produce evidence that variables such as marital status and ‘who brought the participant up’ were independent predictors of CD, initial association of these variables with CD on bivariate analysis perhaps suggests the need for further investigation of this lack of association. Studies have shown that family dysfunction, parental loss and parental deprivation ultimately produced an attendant loss of a sense of submission to authority figures (primarily, the parents) since they were absent [26, 27]. This is because, family is the foundation of human society and is the strongest socializing force of life. “The family teaches children to eschew unacceptable behavior, to delay gratification and to respect the right of others” [26]. Establishments such as schools, religious bodies, and the law enforcement agency, though acting as surrogates, cannot substitute an intact or healthy family [17, 26]. Furthermore, the absence of an intact family, either as a result of dysfunction or loss, (by death or divorce/separation) may lead to emotional and material deprivation.

In many African cultures, whenever there is divorce or separation, the mother is often the one to leave the house regardless of the children’s interests [27]. These children are sometimes left to cater for themselves and the very absence of material resources may lead them into wandering in order to beg for alms or resorting to stealing as means of surviving.

More importantly, the age at which a parent is lost is very crucial and significantly related to developing conduct behaviors [23]. Indeed this variable demonstrated a trend of significance in the regression model (p = 0.065; OR 3.259; CI 0.927–11.450) and was clearly significant in the bivariate analysis with a significantly higher proportion of these incarcerated adolescents who met the criteria for conduct disorder, reporting losing at least one of their parents before their fifth birthday (*χ*^2^ = 4.362, p = 0.037). This age coincides with the period when a child internalizes the family values and develops a conscience, i.e., the “superego” [2]. It has been proposed to serve as “agency that provides ongoing scrutiny of a person’s behavior, thought, and feelings; makes comparison with expected standards of behavior and offers approval or disapproval” [2]. A defect in the establishment of conscience or in internalizing the family values may be responsible for the development of delinquency in various ways which include; loss of a sense of submission to authority figures including parents and loss of social control, which could eventually give way to frank criminal behavior. Moreover, it had been reported that living with other relatives for up to 1 year before primary school age is associated with delinquency [23].

Notwithstanding that family setting does not explain any variance in the regression model (OR 0.869; p = 0.852; 95 % CI 0.199–3.793), a signifiant proportion (68 %) of those who met the criteria for conduct disorder were from polygamous homes (*χ*^2^ = 5.276; p = 0.022).

Polygamy, which is a common practice in Africa [28] and many Islamic states [12], is often characterized by adverse conditions such as; unhealthy rivalry among co-wives and among half siblings, most often as a result of disproportionate display of attention or unequal distribution of resources. These may eventually lead to family breakdown and resultant departure of children from such homes to the street where they are exposed to bad influences leading to delinquency.

In Nigeria, family setting (monogamous or polygamous) is a major determinant of the family size, with polygamous families being more likely to be larger than monogamous ones. Previous authors have technically defined large sibship as having five or more siblings [12, 29] and this definition was adopted for the purpose of this study. In this setting, our observation reveals that, family size is 4.6 times as important as family setting in predicting CD, hence individuals from homes with large sibship are 4.6 times more like to develop CD than those from homes with small sibship (OR 4. 630; 95 % CI 1.433–14.964).

Large sibship, which may be a function of polygamous family setting, is often uncommon in most developed world, where polygamy is either not encouraged by the law or not a usual practice. This could largely explain the reason why this variable was not explored by most of the studies from Europe and America [4, 6, 7, 30]. Nevertheless, our study agrees with those of some authors [16, 17], who found an association between large family and conduct behaviors.

Among these people, conduct behaviors may have been caused by the dilution of family resources such as funds and accommodation, where finite family resources are allocated thinly across a larger number of children. The inadequate resources may not only be in form of monetary or material possessions, but lack of other nonmaterial resources such as adequate time and attention spent on each child, helping them with schoolwork, providing encouragement and positive reinforcement. These nonmaterial resources are equally crucial for children’s development, because, they are positively related to their moral and social development [17, 28].

Finally, another variable with significant contribution to the prediction of CD was ‘Previous history of incarceration’ (p = 0.043). Juveniles who were repeatedly incarcerated were about five times more likely to develop CD OR 4.99; 95 % CI (1.048–23.846).

This is consistent with previous studies [12, 14, 19] which have indicated that a significant proportion of those with CD demonstrated some level of recidivism. This may further support an earlier argument that a mental disorder of a behavioral type was a major contributor to juvenile re-offending and such antisocial trends could continue except affected adolescent offenders were systematically investigated and properly managed [4, 5, 9, 11]. As opined by Alemika and Chukwuka [15], in their study among juvenile offenders in Nigeria, more efforts are being channeled towards keeping young offenders/disturbed adolescents off the community rather than rehabilitating them. Most often these efforts only incubate more delinquent behaviors, rather than properly addressing them. Some of these incarcerated but ‘untreated’ juveniles are even introduced to substance use [2, 19, 20, 31] and more dangerous vices while in incarceration [15]. This may explain why they go back into offending or sometimes become more hardened, leading to more serious offences. Moreover, about 97 (66.0 %) of these incarcerated adolescents, who were in for noncriminal reasons, which included, wandering and labelled, ‘beyond parental control’ stand the risk of being exposed to criminal vices. Nevertheless, the meta-analysis of DeSwart et al. had clearly indicated that the implementation of evidence-based treatment in residential care could have a more positive effect on behavioral problems [23]. This is however challenging to the low or middle income countries such as Nigeria, where there are no separate facilities for the juvenile offenders and those merely in need of protection [5, 15].

A modest proportion of the variance (23.6 %) in CD occurrence was explained by the two independent predictors, which perhaps suggest that other factors related to the development of CD were not investigated in this study. These factors include some biological and other psychological factors which may form the subject for further research.

### Recommendations

Based on our study findings, there is the need for well-planned psychosocial interventions for adolescent offenders rather than mere incarceration.

On the basis of these, we therefore recommend a collaboration between the mental health providers and the juvenile justice system in adopting a more productive interventional method in handling disturbed children. It is equally important to develop social support strategies as is obtainable elsewhere in the world [4, 32], for children with dysfunctional family. These strategies should involve comprehensive early interventions such as improved parental supervision which will serve potent preventative functions.

Furthermore, more intensive media programs, in creating awareness and training of parents on early identification of this disorder are recommended. This approach has been shown to be an effective way of reducing conduct problems before antisocial behavior crystallizes [11]. Lastly, future research is necessary to investigate how these highlighted risk factors and other psychological/biological factors interact with CD and its severity particularly in Nigeria.

### Limitations

Our study has some important limitations which should be addressed. Since it was not feasible to interview the participants’ parents or care giver, the data obtained were subject to the reliability of participants’ self-report of symptoms. It is therefore not possible to rule out the likelihood of over or under reporting symptoms particularly among those with conduct problems.

The generalizability of our study across gender is clearly limited, owing to the single sex nature of our sample which is a direct result of the legal provisions regarding Borstal Institutions in Nigeria [33].

The cross-sectional nature of this study clearly limits its ability to establish the direction of causality in the relationship between identified risk factors and CD.

### Strengths

Conversely, the strengths of this study lie in its use of an internationally accepted instrument, which has also been used in this environment with comparable results [5] and the use of a larger sample size than had been used in the country by previous researchers [5, 23] as well as robust attempt at clearly delineating specific independent predictors of CD from its myriad risk factors.

## Conclusions

In this study, CD among incarcerated adolescents was about three times as high as in the general population. We also found an association between conduct disorder (CD) and some socio-demographic variable such as older age group; large family size; parental separation especially below the age of 5 years. Nevertheless, the effect of these variable were insignificant in predicting CD after a logistic regression analysis. Large sibship and repeated incarceration were the strongest predictors of CD and so, were the major factors related to the risk of developing CD in this environment.

We recommend a re-direction of mental health policy especially as it affects these disturbed children labeled “juvenile offenders” to break the cycle of recidivism through the composite interplay of justice, mental condition and child welfare.

Unlike in Europe and America where there are provisions for ‘Looked after kids’ [4, 32], i.e., children in public care, children from large family often constitutes those who are called ‘street kids’ in Nigeria [34]. They are mostly from impoverished polygamous homes and are available in every part of the country, especially the urban cities [34]. These individuals are eventually apprehended for wandering and kept in the same facilities for young offenders which are often ill-equipped to effectively cater for their psychosocial needs. They ultimately learn dangerous vices and strengthens the circle of recidivism. Thus, these juveniles may continue to suffer, unless there is a good understanding of the composite interplay among justice, mental condition and child welfare.

In the light of this, we believe that our study will lend more support to the existing ones in advising a redirection of mental health policy especially as it affects these disturbed children labeled “juvenile offenders”.

## Abbreviations

CD: conduct disorder
MINI-KID: Mini International Neuropsychiatric Interview for Children and Adolescents
CI: confidence interval
